# Genetic and Brain Mechanisms of Addictive Behavior and Neuroadaptation

**DOI:** 10.3390/brainsci12010051

**Published:** 2021-12-30

**Authors:** Tamara J. Phillips

**Affiliations:** 1 Department of Behavioral Neuroscience, Oregon Health & Science University, Portland, OR 97239, USA; phillipt@ohsu.edu; 2 Portland Alcohol Research Center, Oregon Health & Science University, Portland, OR 97239, USA; 3 Veterans Affairs Portland Health Care System, Portland, OR 97239, USA

Genetic differences play a role in the susceptibility to addictive drug use, the probability that the use of these drugs will escalate and result in a drug use disorder, and whether relapse to use will occur during or after treatment. These hazards are likely related to differences in the sensitivity to drug effects, such as rewarding and aversive effects, cognitive and social behavioral effects, effects on emotional regulation, and brain neuroadaptive effects. The magnitude of all of these effects may be impacted by genetic differences. In the current Special Issue, we have included 12 articles and two reviews. They cover aspects of the broad subject area of the genetic and brain mechanisms underlying addiction, and include eight papers focused on alcohol (ethanol—EtOH), two on methamphetamine (MA), one on nicotine, one on endocannabinoids, a review that incorporates information about the role of the centrally projecting Edinger–Westphal nucleus in the actions of addictive drugs, and a review focused on the role of a glutamate receptor subtype in the negative affective effects of alcohol.

Two papers describe the utilization of classical genetic behavioral approaches to investigate the genetic relationships between EtOH phenotypes at the behavioral level. Thus, Crabbe et al. [1] addresses whether mice selectively bred for high blood EtOH concentrations, as a result of consuming binge-like levels of EtOH, also exhibit greater or lesser sensitivity to an aversive effect of EtOH, compared to their non-selected counterparts. They find that breeding for high blood EtOH concentrations is associated with reduced sensitivity to EtOH-conditioned aversion. They also report that manipulations to induce even higher EtOH intake in the selected line of mice reduce intake on the following day, potentially due to the achievement of a dose high enough to induce aversion. Cunningham [2] uses a panel of inbred mouse strains to investigate the genetic relationship between a conditioned aversive effect of EtOH and several additional EtOH-related traits. Consistent with Crabbe et al. [1], genotypes that consume more EtOH are generally less sensitive to EtOH-induced conditioned aversion. Overall, the results in these papers are consistent with a body of work suggesting that reduced sensitivity to the aversive effects of EtOH serves as a genetic risk factor for higher EtOH intake.

Classical genetic models are also used in the nicotine- and endocannabinoid-related papers [3,4] in this Special Issue. Akinola et al. [3] uses the approach of studying two highly genetically related substrains of mice. This approach reduces the genetic complexity, so that if phenotypic differences are found, the gene-finding efforts are simplified. Akinola et al. [3] identify strain differences for the effects of both acute and repeated nicotine administration, and also identify nicotine-related traits for which the strains do not significantly differ. The next step would be to perform research designed to identify the underlying genetic variants responsible for the strain differences. Kirchhoff et al. [4] turn to mice that were bidirectionally selectively bred for EtOH preference, and they obtain data that support a role for endocannabinoid systems in stress-related behaviors associated with differences in EtOH intake. The outcomes are complicated and, in some cases, sex dependent, supporting the need for additional research.

Comprehensive genomic approaches are used in three of the papers in this Special Issue [5,6,7]. Hitzemann et al. [5] focus on the genetic factors that impact the susceptibility to voluntarily consuming MA and compare the brain transcriptome of mice that were bidirectionally selectively bred for high vs. low voluntary MA intake. Three brain reward pathway regions are analyzed, with the ventral midbrain being most strongly impacted by selective breeding for MA intake. The results are complex, but among the findings are outcomes implicating glutamate-mediated synaptic plasticity in differential risk. Farris et al. [6] uses a single inbred mouse strain with high propensity to consume EtOH to examine the independent and interactive effects of stress and EtOH on the prefrontal cortex transcriptome. This region was chosen based on evidence of its role in decision making and compulsive EtOH consumption. This paper highlights the importance of exploring changes in the transcriptome over time, which may provide clues to stress- and EtOH-induced neuroadaptations. Finally, Ferguson et al. [7] describe a strategy for using computational approaches to nominate novel treatments for alcohol use disorder. An advantage for this approach is the existence of multiple databases, including the Kyoto Encyclopedia of Genes and Genomes (KEGG), Library of Network-based Cellular Signatures (LINCS L1000), and Connectivity Map (CMap) databases. Of significance is the web-interface tool the authors created for identifying drugs that target specific pathways and for nominating mechanisms of drug action.

Human and preclinical animal studies support the roles of neuroimmune system function and proteins in alcohol and drug use disorders, e.g., [8,9]. Three papers provide additional data in this area. Bajo et al. [10] specifically focus on the myeloid differentiation primary response protein (MyD88), an adaptor protein used by both Toll-like receptors and interleukin-1 receptor pathways, which are hypothesized to contribute to alcohol use disorder. Bajo et al. [10] study MyD88 in relation to GABAergic transmission within the central nucleus of the amygdala, a brain region potentiated by EtOH and modulated by interleukin-1β. Their results indicate that MyD88 modulates the magnitude of EtOH and interleukin-1β effects in this brain region. Kays and Yamamoto [11] study MA-induced neuroinflammation using a comprehensive approach. Gene expression is examined in rat striatal and prefrontal cortex microglia/macrophage cells after in vivo binge exposure to MA. Expression data and Ingenuity Pathway Analysis (IPA, http://www.ingenuity.com accessed on 25 December 2021) provide details of an overall increase in pro-inflammatory state, induced by MA. Finally, Lawrimore et al. [12] use a co-culture approach with murine microglial cells and human neuroblastoma cells to model EtOH induction of immune signaling within and between brain cells. Co-culturing alters the effects of EtOH on specific immune signaling molecules found in single-culture conditions.

The remaining papers and reviews in this Special Issue focus on brain circuitry and brain mechanisms in addiction. Thus, among other changes, Frost et al. [13] find EtOH-induced increases in dendritic length and branching in prelimbic neurons in a preclinical study of rats passively exposed to EtOH by inhalation. As they discuss, the differences in the prelimbic neuron results, compared with the results for the nucleus accumbens core from their prior work, lead them to conclude that neural adaptations to EtOH are regionally distinct. The review by Kasten et al. [14] concludes that there is considerable support for metabotropic glutamate receptor 5 modulation of the negative affective states associated with protracted EtOH withdrawal. The review by Zuniga and Ryabinin [15] describes the literature supporting a role for the centrally projecting Edinger-Westphal nucleus in the sensitivity to EtOH and other addictive drugs, and discusses the involvement of distinct populations of neurons. Finally, the single clinical study described in this Special Issue is by Meyers et al. [16], who combine polygenic risk scores derived from a genome-wide alcohol dependence association study with brain region connectivity data derived from inter- and intra-hemispheric electroencephalogram (EEG) recordings. The EEG data were obtained from adolescent and young adult offspring from the National Institute on Alcohol Abuse and Alcoholism-funded study, known as the Collaborative Study on the Genetics of Alcoholism. A unique finding is that the polygenic risk for alcohol dependence influences EEG coherence—a measure of integration between brain regions. Because this relationship predates heavy alcohol use, EEG coherence could be a risk marker for alcohol use disorder.

The papers in this Special Issue provide new insights into the brain mechanisms of addictive behavior and neuroadaptation. The studies confirm behavioral markers of risk, and nominate new biological and physiological risk markers. Importantly, accumulating data from studies similar to those described here, along with computational approaches that make use of informative databases, see [7], could benefit therapeutic development, which is a major goal of addiction research.
